# Validation and interdevice reliability of a behavior monitoring collar to measure rumination, feeding activity, and idle time of lactating dairy cows

**DOI:** 10.3168/jdsc.2023-0467

**Published:** 2024-03-29

**Authors:** J.V.R. Lovatti, K.A. Dijkinga, J.F. Aires, L.F.C. Garrido, J.H.C. Costa, R.R. Daros

**Affiliations:** 1 Department of Animal and Veterinary Sciences, The University of Vermont, Burlington, VT 05405; 2 Graduate Program in Animal Science, School of Medicine and Life Sciences, Pontifícia Universidade Católica do Paraná, Curitiba, 80215-901, Brazil; 3 Animal Science Department, Federal University of Rio Grande do Sul, Porto Alegre, 91540-000, Rio Grande do Sul, Brazil

## Abstract

Interdevice precision and accuracy are not investigated for precision livestock farming (PLF) technologies, but are fundamental for the use of data in populational metrics and to compare cows' data. This study aimed to validate a behavior monitoring collar (BMC; CowMed, Santa Maria, RS, Brazil) and its interdevice reliability. First, we compared observations with the BMC, and second the interdevice precision and accuracy for rumination, feeding activity, and idle time of lactating dairy cows. Holstein cows (n = 23) were housed in a voluntary milk system freestall barn and fitted with 2 devices within the same cow. Observations were made over 2 periods of one day (0700 to 1100 h, 1400 to 1700 h); the 7 h per cow were summarized for each behavior to assess the agreement of observed behavior and BMC data. To assess the interdevice reliability, 26 d of BMC data were summarized by day per cow for both devices. Pearson correlation (r), coefficient of determination (R^2^), Lin's concordance correlation coefficient (ρ_c_), linear regression, and Bland-Altman plots (BAP) were calculated for each period of observation. For the validation, we found high correlations for feeding activity, very high for idle time, but low correlations for rumination. The BAP were deemed acceptable and without bias; BAP mean differences ± SD were 0.83 ± 4.01, −0.48 ± 4.15, and 7.17 ± 3.94 min/h for rumination, feeding activity, and idle time, respectively. The slope of the linear regression did not differ from 1 for any behaviors but idle. For interdevice comparison, we found moderate correlations for feeding activity and idle time, and a low correlation for rumination. The BAP was deemed acceptable and without bias; BAP mean differences were −0.36 ± 2.84, 0.45 ± 3.51, and −0.06 ± 2.81 min/h for rumination, feeding activity, and idle time, respectively. All slopes of the linear regressions differed from 1 except feeding time. Thus, the interdevice comparison did not meet the accuracy criteria. In summary, this study validated the precision of the BMC for recording feeding activity of lactating dairy cows.

## Highlights

• The BMC was precise when recording feeding activity behavior.
• The BMC interdevice reliability was low when recording dairy cows' behavior.
• The BMC allows behavior monitoring on an individual basis.

## Graphical Abstract

**Figure:**
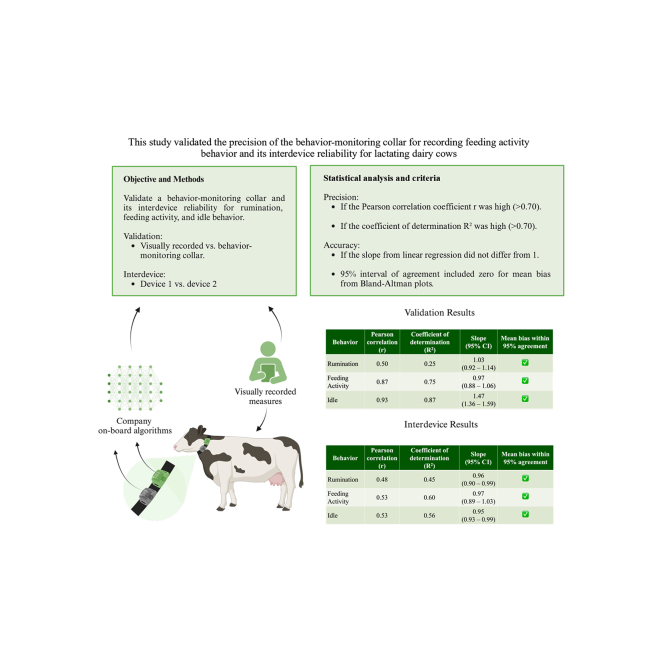
**Summary:** The objective of this study was to validate a behavior-monitoring collar (BMC), comparing first with visual observation and second with interdevice precision and accuracy for rumination, feeding activity, and idle time of lactating dairy cows. A total of 23 Holstein cows were fitted with 2 devices within the same collar and observed over 1 day in 2 periods. This study validates the BMC precision for recording feeding behavior of lactating dairy cows.

Monitoring animal behavior visually is subjective and requires a substantial amount of time (Eerdekens et al., 2021). Precision livestock farming (**PLF**) technologies are a noninvasive, objective measurement of animal behavior using algorithms to process raw data (Costa et al., 2021) and are able to continuously detect real-time behavioral changes (Borchers et al., 2016). Technologies are deemed valid when they achieve satisfactory precision and accuracy compared with a gold standard (Royston and Altman, 2013).

Precision of PLF devices for monitoring cows' behavior has been assessed by Pearson correlation coefficient (r) and Lin's concordance correlation coefficient (ρ_c_) (Bikker et al., 2014; Borchers et al., 2016), or coefficient of determination (R^2^), but very few studies have reported accuracy results (Grinter et al., 2019). Accuracy of PLF devices has been exanimated by using the slope of the regression line and Bland-Altman plots (**BAP**). The BAP are useful to evaluate the bias between the mean differences and to estimate an agreement between 2 methods (Giavarina, 2015). The evaluation of the accuracy is essential, and it represents how closely the measures (i.e., automated recorded behaviors) are to the true values (i.e., observations) (Tedeschi, 2006). Thus, accuracy enables the development of benchmarking, allowing the comparison of the behavior recorded by the PLF device under research or farm conditions.

Despite the popularity of PLF devices, there have been few to no studies investigating interdevice reliability. Interdevice reliability is relevant and should be minimal when comparing data between and within subject (Santos-Lozano et al., 2012). The use of data for populational measurements to make comparisons between subjects is an opportunity for PLF, but it requires interdevice reliability. In sport-tracking devices, interdevice reliability of accelerometers was found to be highly variable (Nicolella et al., 2018). Thus, we suggest that interdevice variability may exist between devices, and it varies depending on the behavior measured. The aim of this study was to validate the device and its interdevice precision and accuracy of a behavior monitoring collar (**BMC**) on lactating dairy cows for ruminating, feeding activity, and idling time. To our knowledge there were no other studies validating interdevice precision and accuracy of a PLF device in commercial settings.

This study was approved by the animal use ethics committee of the Pontifícia Universidade Católica do Paraná (CEUA-PUCPR #02090) and conducted at the Fazenda Experimental Gralha Azul of PUCPR (Fazenda Rio Grande, Paraná, Brazil).

Animals were housed in a freestall barn divided into 2 pens approximately 85 m^2^/pen with a 17-m^2^ feed alley, stocked with approximately 31 cows/pen. Stall stocking density was <100%; stalls were fitted with mattress covered by 2 to 5 cm of sawdust and cleaned daily. The barn was equipped with a voluntary milking system. Cows were fed a partial mixed ration, plus a commercial pellet (approximately 4 kg/d). The mixed ration was formulated following the NRC (2001) recommendations using RLM 3.3 Software (ESALQ-USP, São Paulo, Brazil). The diet was set to meet the requirements of lactating dairy cows producing at least 36 kg of milk/d. Cows were fed twice a day at approximately 0800 and 1600 h and had ad libitum access to fresh water. Sample size was determined following Friedman (1982). Seventeen cows was the minimum number to detect an assumed effect size of 0.70 (r as a measure of effect size) for a correlation as described by Friedman (1982): power of 0.90 and a type I error probability of 0.05 (2-sided). From a herd of 62 dairy cows, 24 Holstein cows (mean ± SD; DIM: 208.78 ± 127.69; parity: 1.3 ± 0.6; and milk yield: 34.88 ± 8.66 kg/d) were selected using the DIM and lactations (primiparous and multiparous) as criteria and were divided into 2 randomly selected groups within pens.

The cows were fitted with a commercially available BMC (CowMed, Santa Maria, RS, Brazil) 1 wk before observation started as recommended by the manufacturer as the adaptation period. The BMC consists of a device (11.5 × 7 × 3.3 cm; 140 g) + nylon band (120 g) + counterweight (240 g). The life expectancy of batteries for the BMC is up to 5 yr. The BMC data were wirelessly transmitted hourly to a base station connected to the internet placed inside the barn. The barn base station was able to store data for up to 24 h. All BMC devices were synchronized to a local hour (GMT-03). Each enrolled cow had 2 devices within the same collar positioned longitudinally in the middle of the left side of the collar near the animal's ear. The BMC uses a preprocessing data mechanism where the data are recorded by minute but encoded in 1-h bouts (i.e., the data cloud received the data in minutes per hour for each behavior [rumination, feeding activity, and idle time]).

The observations were made in 2 periods (0700 to 1100 h and 1400 to 1700 h) within a 24-h time frame to attempt to record a range of behaviors from diurnal variation (DeVries et al., 2003). To match the BMC data recording scheme, the observers were trained to scan sample the focus cows every minute with the aid of smartphones synchronized to the same local hour (GMT-03). Five observers were trained to observe rumination, feeding activity, and idle behavior according to the following ethogram: rumination (regurgitation and re-mastication of a bolus with a rhythmic jaw movement), feeding activity (cow with muzzle in contact with feed, including sorting, smelling, and chewing feed nonstop for ≥5 s; drinking and ingesting mineral), idle (included lying and standing behavior and activities such as walking, grooming, licking, rubbing, and interacting with other cows).

Each observer recorded the same 4 cows at a time during all the observation periods. The inter-rater reliability was assessed through Cohen's weighted kappa weighted equally, and each observer was compared in pairs against a standard rater (Hallgren, 2012). Kappa coefficient was computed separately for each behavior. Inter-rater reliabilities for each observer compared with a standard rater were all above 0.95.

To compare the BMC data with the observers, a total of 19 cows were observed within one experimental day. The observer was positioned within a clear field of view of the focal cow to ensure the constant view of the animal's head and muzzle, without interfering with the cow's behavior. The total time for each cow was summed for each behavior (rumination, feeding activity, and idle) per hour and then summed to the total observed period to assess the agreement of observed behavior to BMC data.

For interdevice comparison, a total of 23 cows were recorded for 26 d; however, the first 3 d, referred to as the synchronization period, and the last day during which collars were detached were deleted from the dataset to avoid unmatched 24-h time-frame data. Recorded data from both devices were summed by day to obtain the total time recorded per day for each behavior. Although the data were extracted in a 60-min block for research purposes, the technology only outputs a daily summary for producers and consultants. In fact, utilization of the daily summary is commonly used in decision-making tools for estrus detection (Mayo et al., 2019) and early disease detection such as for mastitis (Rial et al., 2023) and respiratory diseases (Costa et al., 2021). Daily summarization is important because although external signs of disease or estrus may be a meaningful indication, behaviors such as rumination, feeding activity, and idle may not be meaningful if not observed within an extended time frame (Cantor et al., 2022a). Thus, data were summarized and analyzed by day to be applicable in the field. There was only one BMC failure during the study period, and data were deleted to avoid unmatched data.

Precision was analyzed by a Pearson r and R^2^ with cow as a random effect in the linear regression model, and interpreted following Hinkle (1988): 0.00 to 0.30 = negligible; 0.30 to 0.50 = low; 0.50 to 0.70 = moderate; 0.70 to 0.90 = high; and 0.90 to 1.00 = very high. Additionally, the ρ_c_ was calculated for all behaviors following Lin (1989) and interpreted following McBride (2005): <0.90 = poor; 0.90 to 0.95 = moderate; 0.95 to 0.99 = substantial; >0.99 = almost perfect. Linear regressions were used to calculate the R^2^ and the slope of the relationship between the observations and the BMC and interdevice measures. The BMC was considered precise if the r and R^2^ were high (>0.70). For validation of the BMC against the observations, r and ρ_c_ were analyzed across all cows. For the interdevice comparison, to observe the individual variation over the days within the experimental population, r and ρ_c_ were analyzed for each cow and reported as the median value for the experimental population.

The slope of the regression and BAP (Bland and Altman, 1986) was used to assess the accuracy for each behavior. Bland-Altman statistical results were used to obtain the mean differences of the plots. The BMC was considered accurate if the slope from the linear regressions did not differ significantly from 1 and if the 95% interval of the agreement included zero for mean bias from the BAP. All statistical analyses were performed in R, version 4.1.3 (https://www.r-project.org/).

Descriptive analyses for data observed and BMC are presented in Table 1. For the validation comparisons, the Pearson r was 0.50, 0.87, and 0.93 (*P* = 0.03) for rumination time, feeding activity time, and idle time, respectively. The R^2^ was 0.25, 0.75, and 0.87 (*P* = 0.03) for rumination time, feeding activity time, and idle time, respectively. Also, Lin's ρ_c_ was 0.48, 0.86, and 0.63 for rumination time, feeding activity time, and idle time, respectively. Slopes of linear regressions for observations versus BMC did not differ significantly from 1 except for idle behavior. The slope of regression used to assess accuracy for observations compared with BMC was found to be 1.03 (95% CI: 0.92–1.14; *P* < 0.001) for rumination time, 0.97 (0.88–1.06; *P* < 0.001) for feeding activity time, and 1.47 (1.36–1.59; *P* < 0.001) for idle time. The BAP was used to assess the bias between the mean difference of observations and BMC, and the agreement interval, for rumination (Figure 1A), feeding activity (Figure 1B), and idle (Figure 1C). The BMC was found to have most cows within the 95% interval of agreement of the BAP; only 2 cows were outside this interval for feeding activity. Also, all of the BAP included zero within the CI for observations compared with BMC. Mean differences were used to determine whether one measure was over- or underestimating another. The results of the mean difference between observations and BMC were rumination time: 0.83 ± 4.01, feed activity time: −0.48 ± 4.15, and idle time: 7.17 ± 3.94 min/h.

**Table 1:** Mean ± SD, minimum, and maximum time in minutes per hour of lactating dairy cows spent ruminating, feeding, and idling, as observed and as recorded by both behavior-monitoring collars (BMC1 and BMC2), with the percentage of time spent displaying the corresponding behavior observation in parentheses

Item	Observed		BMC1		BMC2	
	Mean ± SD (% ± SD)	Minimum/maximum	Mean ± SD (% ± SD)	Minimum/maximum	Mean ± SD (% ± SD)	Minimum/maximum
Ruminating (min/h)	18.9 ± 4.3	12.1 (20.2%)/	17.5 ± 3.1	11.8 (19.7%)/	17.9 ± 3.2	10.9 (18.1%)/
	(31.5 ± 7.2%)	28.3 (47.1%)	(29.2 ± 5.1%)	21.2 (35.3%)	(29.8 ± 5.3%)	22.8 (37.9%)
Feeding activity (min/h)	20.0 ± 8.2	1.7 (2.9%)/	13.2 ± 4.2	7.9 (13.2%)/	12.8 ± 4.3	7.8 (13.1%)/
	(33.3 ± 13.7%)	31.3 (52.1%)	(22.1 ± 7.0%)	23.1 (38.4%)	(21.3 ± 7.2%)	27.2 (45.3%)
Idle (min/h)	21.2 ± 9.3	8.7 (14.5%)/	18.3 ± 3.5	10.2 (17.0%)/	18.3 ± 4.0	10.3 (17.2%)/
	(35.3 15.5 ± %)	43.9 (73.1%)	(30.4 ± 5.8%)	23.1 (38.5%)	(30.5 ± 6.6%)	25.1 (41.9%)

**Figure 1:**
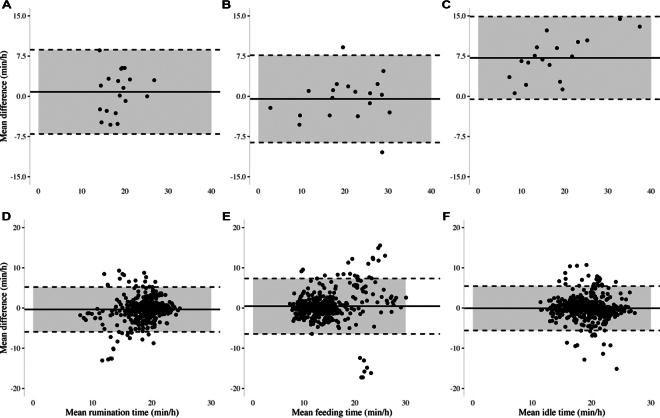
Bland-Altman plots illustrating agreement between the differences in observations and the behavior-monitoring collar (BMC) measures for rumination (A), feeding activity (B), and idle (C) time; and the agreement between the differences in both BMC (BMC1 − BMC2) for rumination (D), feeding activity (E), and idle (F) time. The solid line indicates the mean difference between the measures, and the dotted lines represent the SD from the mean difference. The x-axis represents the range of the mean values between the measures. The y-axis represents the difference between the measures.

For interdevice measures summarized by total time recorded per day for each of the 23 cows over 22 d, the Pearson r [median (first and third quartiles, **Q1**, **Q3**)] was 0.48 (0.29, 0.63), 0.53 (0.40, 0.72), and 0.53 (0.33, 0.79) for rumination, feeding time, and idle time, respectively (Figure 2). The R^2^ was 0.45, 0.60, and 0.56 (*P* < 0.01) for rumination time, feeding activity time, and idle time, respectively. The ρ_c_ [median (Q1, Q3)] for interdevice measures was 0.38 (0.16, 0.60), 0.50 (0.13, 0.64), and 0.40 (0.24, 0.75) for rumination time, feeding time, and idle time, respectively. The BAP was used to assess the bias between the mean difference of BMC and the agreement interval for rumination (Figure 1D), feeding activity (Figure 1E), and idle (Figure 1F). For interdevice comparison, all slopes differed from 1 except for feeding time. The slope was found to be 0.96 (95% CI: 0.90–0.99; *P* = 0.02) for rumination time, 0.97 (0.89–1.03; *P* = 0.26) for feeding time, and 0.95 (0.93–0.99; *P* = 0.01) for idle time. The BMC were found to have most cows within the 95% interval of agreement of the BAP. Also, all the BAP included zero within the interval of agreement for interdevice comparisons. Additionally, the results of the mean differences between BMC were rumination time: −0.36 ± 2.84, feeding activity time: 0.45 ± 3.51, and idle time: −0.06 ± 2.81 min/h.

**Figure 2:**
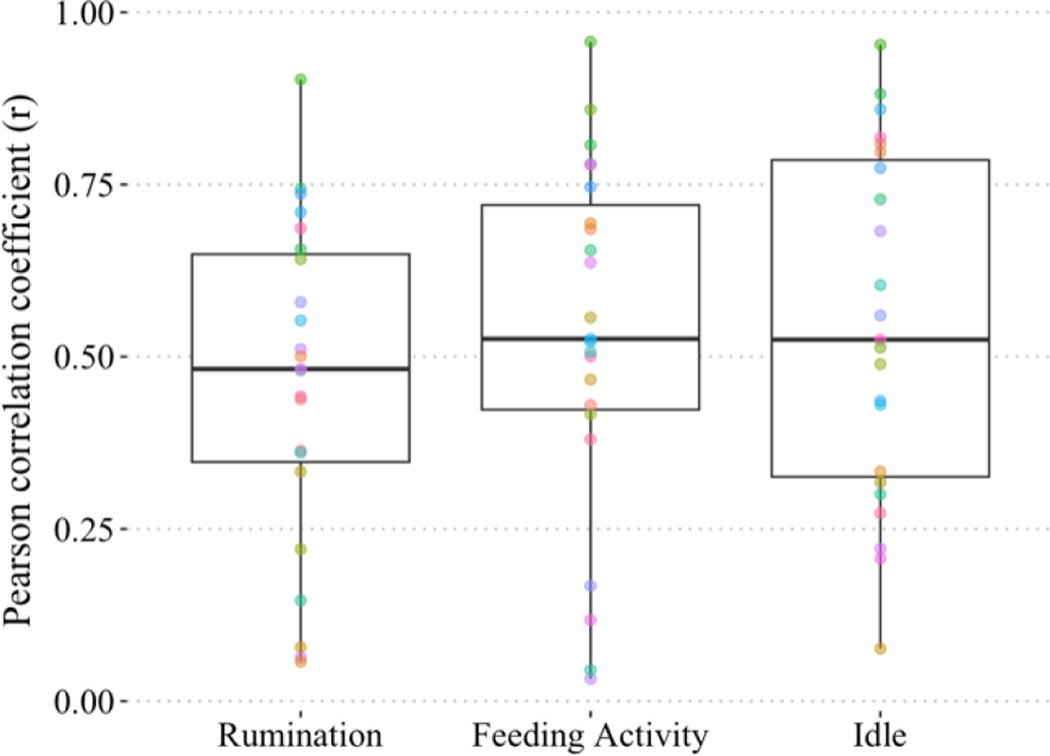
Boxplots of the Pearson correlations for interdevice comparisons (BMC1 vs. BMC2) for rumination, feeding activity, and idle behavior of 23 Holstein dairy cows within a 24-h time frame over 22 d. The median (50th percentile) is represented by the heavy dark line within each box; lower and upper lines of the box represent the 25th and 75th percentiles, respectively. Whiskers extend to the smallest and largest nonoutlier statistical values. Each dot represents an individual cow.

The BMC used in this study showed a high correlation between the BMC compared with a trained observer for feeding activity and idle behavior, but low correlations for rumination. Studies that validated other similar commercial monitoring behavior devices found comparable results to this BMC. Bikker et al. (2014), studying freestall-housed dairy cows, had very high correlations for feeding and idle time. Borchers et al. (2016), when validating PLF devices using freestall-housed dairy cows, had very high results for feeding behavior. Grinter et al. (2019) validated a very similar device under similar conditions to those of this study, showed very high results for ruminating, feeding, and resting behaviors. Overall, we deemed the BMC assessed in this study precise to measure feeding activity when compared with observations, but more refinements are needed to precisely monitor ruminating and idle time.

Accuracy has been assessed in validation studies by analyzing the slope of the regression line (Chizzotti et al., 2015; Grinter et al., 2019) and the BAP (Cantor et al., 2022b; Renaud et al., 2022) to assess the agreement between 2 measures. Previous research suggested that accuracy is not only important for helping farmers to monitor dairy herds in real time, but also allows data to be compared across farms (Grinter et al., 2019). Although all BAP satisfied accuracy requirements, the slope of the regression line for idle time showed that the BMC overestimates idle behavior when compared with observations. We may visually assess in the BAP (Figure 1C) where the 0 was found to be close to the lower limit of agreement, meaning a tendency to overestimating the idle time, even though all the cows were encompassed within the 95% limit of agreement. The overestimation of idle behavior may be attributed to open-set recognition, where different activities are misclassified into known activities on algorithms trained for a limited set of behaviors (Mao et al., 2023). The BMC may account for walking, standing, lying, and other activities not identified as rumination or feeding activity within the idle behavior, thus resulting in a difference between the observations and BMC. However, BAP define the intervals of agreements and do not state whether those limits of agreement are acceptable or not (Giavarina, 2015). Thus, it is essential to take into consideration the biological aspect of the variables investigated. Future research should investigate and clarify the factors that affect the accuracy of PLF devices.

There is a lack of discussion regarding interdevice reliability in PLF devices, and to our knowledge, this is the first study investigating the interdevice precision and accuracy of a BMC for lactating dairy cows. Interdevice reliability is directly correlated with the accuracy of the data recorded. Low reliability may lead to inaccurate measures, affecting the detection of abnormal cow behavior. Pearson correlation and R^2^ did not meet the criteria of precision for the interdevice comparison of the BMC in this study. Furthermore, the BMC did not meet all of the accuracy criteria, but no bias was observed when evaluating the data obtained from interdevice comparison. However, an increase in variation as the time increased was observed for feeding activity time (Figure 1E). In a recent study by Benaissa et al. (2023), the integration of data collected from accelerometers and ultra-wideband location devices yielded improved outcomes for feeding and ruminating time when compared with the utilization of accelerometer data alone. The context-aware modeling, such as location, enables accurate categorization of behaviors, suggesting a prospective future approach for enhancing the accuracy of the BMC investigated in our current study.

Studies investigating triaxial accelerometer interdevice reliability and factors affecting data collection demonstrated high reliability between devices exposed to different applications (veterinary use [Martin et al., 2017]; human health and activity [Takacs et al., 2014; Dontje et al., 2015; Nickerson et al., 2020]). Overall, triaxial accelerometer devices proved to have high reliability between devices in different applications; thus, they may be applied to monitor cows' behavior such as rumination, feeding activity, and idle time. Nevertheless, we deemed the BMC to have low accuracy when comparing both devices measuring rumination, feeding activity, and idle time. Despite low accuracy, on-field applications are based on individual machine learning, and thus the BMC is applicable for monitoring animal behavior individual data and detecting temporal variability once the algorithm evaluates each cow's average daily rate of acceleration and creates a behavior index. However, between animals, in which accuracy is demanded, future investigations are needed to improve the reliability of the BMC.

There are some limitations to be considered when evaluating the data obtained in this study. The objective of this study was to validate the BMC and its interdevice reliability in lactating dairy cows. Interactivity similarity, which is a case where different animal behaviors have similar characteristics or movement patterns (Mao et al., 2021), such as panting and licking, may result in interference of behavior detection by the BMC. Thus, the algorithm may have classified other behaviors as one of the behaviors of interest in this study. Furthermore, this is an independent validation of the algorithm, and likely the ethogram employed in this investigation may exhibit discrepancies in comparison to the ethogram that served as the basis for developing the BMC algorithm. Future research should investigate factors affecting the validity of PLF devices analyzing larger datasets to understand the magnitude of the variability.

This study evaluated the precision, accuracy, and interdevice reliability of a commercially available BMC. Feeding activity was found to be highly correlated with observations deeming the device useful to measure feeding activity autonomously. However, although the BMC allows the collection of constant and consistent data on an individual basis, it still lacks accuracy.

## References

[bib1] Benaissa S., Tuyttens F.A.M., Plets D., Martens L., Vandaele L., Joseph W., Sonck B. (2023). Improved cattle behaviour monitoring by combining Ultra-Wideband location and accelerometer data. Animal.

[bib2] Bikker J.P., van Laar H., Rump P., Doorenbos J., van Meurs K., Griffioen G.M., Dijkstra J. (2014). Technical note: Evaluation of an ear-attached movement sensor to record cow feeding behavior and activity. J. Dairy Sci..

[bib3] Bland J.M., Altman D. (1986). Statistical methods for assessing agreement between two methods of clinical measurement. Lancet.

[bib4] Borchers M.R., Chang Y.M., Tsai I.C., Wadsworth B.A., Bewley J.M. (2016). A validation of technologies monitoring dairy cow feeding, ruminating, and lying behaviors. J. Dairy Sci..

[bib5] Cantor M.C., Casella E., Silvestri S., Renaud D.L., Costa J.H.C. (2022). Using machine learning and behavioral patterns observed by automated feeders and accelerometers for the early indication of clinical bovine respiratory disease status in preweaned dairy calves. Front. Anim. Sci..

[bib6] Cantor M.C., Goetz H.M., Beattie K., Renaud D.L. (2022). Evaluation of an infrared thermography camera for measuring body temperature in dairy calves. JDS Commun..

[bib7] Chizzotti M.L., Machado F.S., Valente E.E.L., Pereira L.G.R., Campos M.M., Tomich T.R., Coelho S.G., Ribas M.N. (2015). Technical note: Validation of a system for monitoring individual feeding behavior and individual feed intake in dairy cattle. J. Dairy Sci..

[bib8] Costa J.H.C., Cantor M.C., Neave H.W. (2021). Symposium review: Precision technologies for dairy calves and management applications. J. Dairy Sci..

[bib9] DeVries T.J., Von Keyserlingk M.A.G., Beauchemin K.A. (2003). Short communication: Diurnal feeding pattern of lactating dairy cows. J. Dairy Sci..

[bib10] Dontje M.L., De Groot M., Lengton R.R., Van Der Schans C.P., Krijnen W.P. (2015). Measuring steps with the Fitbit activity tracker: An inter-device reliability study. J. Med. Eng. Technol..

[bib11] Eerdekens A., Deruyck M., Fontaine J., Martens L., De Poorter E., Plets D., Joseph W. (2021). A framework for energy-efficient equine activity recognition with leg accelerometers. Comput. Electron. Agric..

[bib12] Friedman H. (1982). Simplified determinations of statistical power magnitude of effect and research sample size. Educ. Psychol. Meas..

[bib13] Giavarina D. (2015). Understanding Bland Altman analysis. Biochem. Med. (Zagreb).

[bib14] Grinter L.N., Campler M.R., Costa J.H.C. (2019). Technical note: Validation of a behavior-monitoring collar's precision and accuracy to measure rumination, feeding, and resting time of lactating dairy cows. J. Dairy Sci..

[bib15] Hallgren K.A. (2012). Computing inter-rater reliability for observational data: An overview and tutorial. Tutorials in Quantitative Methods for Psychology.

[bib16] Hinkle D.E. (1988).

[bib17] Lin L.I. (1989). A concordance correlation coefficient to evaluate reproducibility. Biometrics.

[bib18] Mao A., Huang E., Gan H., Parkes R.S.V., Xu W., Liu K. (2021). Cross-modality interaction network for equine activity recognition using imbalanced multi-modal data. Sensors (Basel).

[bib19] Mao A., Huang E., Wang X., Liu K. (2023). Deep learning-based animal activity recognition with wearable sensors: Overview, challenges, and future directions. Comput. Electron. Agric..

[bib20] Martin K.W., Olsen A.M., Duncan C.G., Duerr F.M. (2017). The method of attachment influences accelerometer-based activity data in dogs. BMC Vet. Res..

[bib21] Mayo L.M., Silvia W.J., Ray D.L., Jones B.W., Stone A.E., Tsai I.C., Clark J.D., Bewley J.M., Heersche G. (2019). Automated estrous detection using multiple commercial precision dairy monitoring technologies in synchronized dairy cows. J. Dairy Sci..

[bib22] McBride, G. B. 2005. A proposal for strength-of-agreement criteria for Lin's concordance correlation coefficient. NIWA Client Report: HAM2005–062.

[bib23] Nickerson B.S., Medrano N.F., Perez G.L., Narvaez S.V., Carrillo J., Duque M. (2020). Inter-device reliability of wearable technology for quantifying jump height in collegiate athletes. Biol. Sport.

[bib24] Nicolella D.P., Torres-Ronda L., Saylor K.J., Schelling X. (2018). Validity and reliability of an accelerometer-based player tracking device. PLoS One.

[bib25] NRC (2001).

[bib26] Renaud D.L., Hare K.S., Wood K.M., Steele M.A., Cantor M.C. (2022). Evaluation of a point-of-care meter for measuring glucose concentrations in dairy calves: A diagnostic accuracy study. JDS Commun..

[bib27] Rial C., Laplacette A., Caixeta L., Florentino C., Peña-Mosca F., Giordano J.O. (2023). Metritis and clinical mastitis events in lactating dairy cows were associated with altered patterns of rumination, physical activity, and lying behavior monitored by an ear-attached sensor. J. Dairy Sci..

[bib28] Royston P., Altman D.G. (2013). External validation of a Cox prognostic model: Principles and methods. BMC Med. Res. Methodol..

[bib29] Santos-Lozano A., Torres-Luque G., Marín P.J., Ruiz J.R., Lucia A., Garatachea N. (2012). Intermonitor variability of GT3X accelerometer. Int. J. Sports Med..

[bib30] Takacs J., Pollock C.L., Guenther J.R., Bahar M., Napier C., Hunt M.A. (2014). Validation of the Fitbit One activity monitor device during treadmill walking. J. Sci. Med. Sport.

[bib31] Tedeschi L.O. (2006). Assessment of the adequacy of mathematical models. Agric. Syst..

